# Genomic integration of transgenes using UV irradiation in *Pristionchus pacificus*

**DOI:** 10.17912/micropub.biology.000576

**Published:** 2022-05-29

**Authors:** Güniz Göze Eren, Marianne Roca, Ziduan Han, James W Lightfoot

**Affiliations:** 1 Max Planck Research Group Genetics of Behavior, Max Planck Institute for Neurobiology of Behavior – caesar, Bonn, Germany.; 2 Department for Integrative Evolutionary Biology, Max Planck Institute for Biology, Tuebingen, Germany

## Abstract

Transgenes are widely used throughout molecular biology for numerous applications. In *Caenorhabditis elegans,* stable transgenes are usually generated by microinjection into the germline establishing extrachromosomal arrays. Furthermore, numerous technologies exist to integrate transgenes into the *C. elegans* genome. In the nematode *Pristionchus pacificus,* transgenes are possible, however, their establishment is less efficient and dependent on the formation of complex arrays containing the transgene of interest and host carrier DNA. Additionally, genomic integration has only been reported via biolistic methods. Here we describe a simple technique using UV irradiation to facilitate the integration of transgenes into the *P. pacificus* genome.

**Figure 1. Integration of transgenes into the P. pacificus genome:**
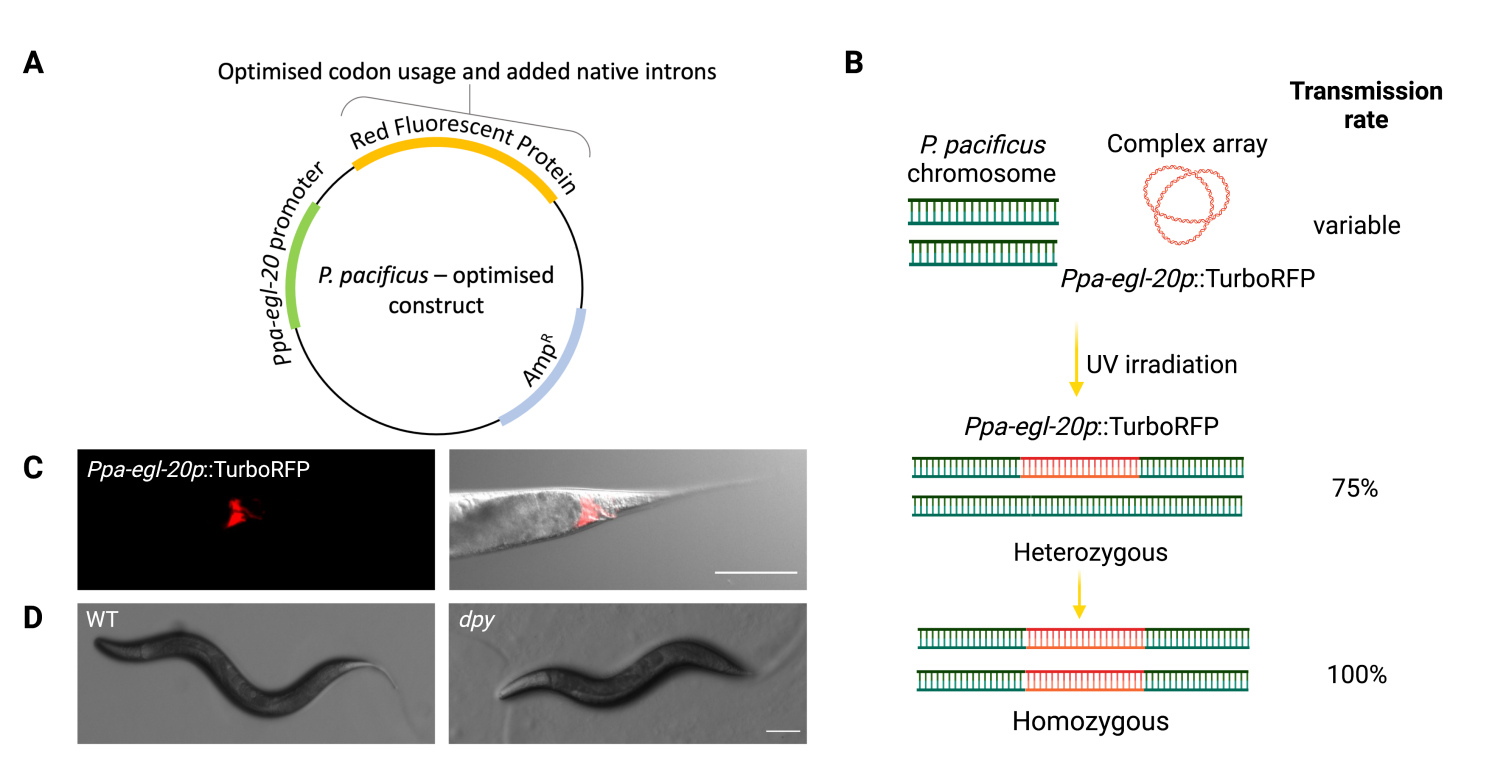
(A) Plasmid carrying *Ppa-egl-20p* ::TurboRFP including codon optimisation and the insertion of native *P. pacificus* introns. (B) Cartoon schematic of UV induced integration methodology. Successful integration occurred in 2 / 250 animals after 0.050 J / cm ^2^ UV exposure increasing transmission to 100%. (C) Confocal image of *Ppa-egl-20p* ::TurboRFP with expression in several tail neurons in a successful UV integrated line. Scale bar = 25 µm. (D) Wild-type *P. pacificus* and an uncloned *Ppa-dpy* mutant generated by UV irradiation and identified in our screen. Scale bar = 250 µm.

## Description

The free-living nematode *Pristionchus pacificus* has emerged as an important system for comparative studies with *Caenorhabditis elegans* . Both nematodes share many common features, however, they also exhibit fundamental differences in many aspects of their biology. This has led to the establishment of *P. pacificus* as an important model for investigating the evolution of diverse traits. These include understanding the evolution of basic cellular processes (Valfort et al. 2018; Rillo-Bohn et al. 2021), the evolution of developmental mechanisms including phenotypically plastic traits (Ragsdale et al. 2013; Werner et al. 2017; Bui et al. 2018; Casasa et al. 2020; Sieriebriennikov et al. 2020), the evolution of chemical signalling systems (Bose et al. 2014; Dong et al. 2020), the evolution of neuronal organisation and connectivity, (Bumbarger et al. 2013; Hong et al. 2019) as well as the evolution of behaviours encompassing feeding, environmental sensing, predation and kin-recognition (Moreno et al. 2017; Lightfoot et al. 2019; Carstensen et al. 2021; Ishita et al. 2021; Lightfoot et al. 2021; Han et al. 2022; Lo et al. 2022; Quach and Chalasani 2022). Correspondingly, a panoply of molecular techniques have been developed to facilitate these studies (Dieterich et al. 2008; Witte et al. 2015; Rödelsperger et al. 2017) including the establishment of transgenic tools (Schlager et al. 2009; Han et al. 2020).

In *P. pacificus* , transgenic methods are well established and share similarities with *C. elegans* in that they are dependent on microinjecting DNA into the cytoplasm of the gonad syncytial. This DNA is taken up by the developing gametes and passed onto subsequent generations (Schlager et al. 2009; Han et al. 2020). Differing from *C. elegans,* however, transgenes in *P. pacificus* are dependent on the formation of complex arrays including a combination of a linearised reporter gene of interest along with restriction digested host carrier DNA. Furthermore, the generation of transgenic lines is less efficient than in *C. elegans* and frequently highly variable transmission rates between lines are observed. Moreover, genomic integration of transgenes has proven especially problematic with successful integrations so far only reported consistently using biolistic methods (Namai and Sugimoto 2018). Here we provide an alternative method for the integration of transgenic elements in *P. pacificus* .

We utilised a previous protocol employing UV irradiation to integrate extrachromosomal arrays in *C. elegans* (Mariol et al. 2013) to instead integrate complex arrays in *P. pacificus* . We firstly generated a strain carrying a complex array consisting of genomic carrier DNA and the *Ppa-egl-20p* ::TurboRFP fluorescent marker (Fig. 1A). This was achieved using well-established microinjection methods (Schlager et al. 2009; Han et al. 2020; Nakayama et al. 2020; Hiraga et al. 2021) resulting in a line with a variable *Ppa-egl-20p* ::TurboRFP transmission rate of around 32%. Subsequently, 20 *Ppa-egl-20p* ::TurboRFP fluorescent animals were each picked onto 10 NGM plates and were exposed to UV irradiation at three different intensities, 0.012 J / cm ^2^ , 0.025 J / cm ^2^ and 0.050 J / cm ^2^ . After irradiation, plates were placed in a 20°C incubator for 3-4 days until the progeny was old enough to screen for fluorescent animals. These F1 fluorescent animals were singled out onto 100-250 individual culture plates per UV energy intensity and the F2 progeny screened for possible integration events. At the highest UV intensity tested (0.050 J / cm ^2^ ), we detected an increase in *Ppa-egl-20p* ::TurboRFP transmission to ≥75% in 2 / 250 lines, indicating the potential successful heterozygous integration of the complex array into the *P. pacificus* genome (Fig 1B). Indeed, 25 % of the F3 animals demonstrated robust neuronal tail expression associated with *Ppa-egl-20p* ::TurboRFP with 100% transmission confirming integration of a complex extrachromosomal array via UV irradiation for the first time in *P. pacificus* (Fig. 1C). Moreover, the expression pattern was also more stable and less variable after integration. Notably, while we have so far only observed successful integrations at the highest UV intensity tested, morphological mutations such as *dumpy* mutants were generated at all UV exposures indicating the induction of genomic lesions (Fig. 1D). Therefore, outcrossing to remove any unintended mutations before further characterisation is essential. Importantly, the presence of mutations at all intensities indicates that integration may also be possible at lower energies. This will require further experimental validation in the future.

Until now, transgenes in *P. pacificus* were frequently maintained indefinitely on complex extra-chromosomal arrays. This can require multiple rounds of injection to acquire lines of sufficiently high transmission for subsequent experimental analysis and strain maintenance can be time-consuming (Schlager et al. 2009; Han et al. 2020). Moreover, the variable transmission rates in non-integrated lines can significantly reduce the potential for further forward genetic approaches such as suppressor screens. Alternatively, biolistic methods have been used to integrate transgenes into the *P. pacificus* genome however, this requires a more costly experimental setup, as well as many additional reagents (Namai and Sugimoto 2018). In comparison, once the initial complex array is established, genomic integration via UV irradiation requires only a UV crosslinker and standard worm culture reagents. Furthermore, screening is fast and simple with the possibility to generate many independent lines. Therefore, this method should facilitate the rapid generation of new lines expressing integrated transgenes for the *P. pacificus* community. In the future, the development of more sophisticated and specific integration methods such as *Mos* based technologies (Frøkjær-Jensen et al. 2014) should be a priority for *P. pacificus* .

## Methods

**Worms strain maintenance**

All worms were maintained at 20°C on NGM plates seeded with *Escherichia coli* OP50. Strains used are found under reagents below.

**Plasmids**

The plasmid pZH009 containing the codon optimised *Ppa-egl-20p* ::TurboRFP with *P. pacificus* specific introns from *Ppa-rps-1* was selected for integration. Further Information can be found in the reagents section below as well as the full plasmid sequence in Han et al (2020).

**Imaging methods**

Worms were imaged on a Zeiss Axio Zoom v.16 with zen software and an Olympus FV 1000 confocal microscope.

**Protocol for generating UV integrated transgenic lines in *P. pacificus***

*Preparing injection mix and microinjection*

*1.* Grow *E. coli* bacteria containing the plasmid of choice (we used pZH009 containing *Ppa-egl-20p* ::TurboRFP) overnight and subsequently extract plasmids via miniprep kit as per the manufacturer’s guidelines (Qiagen).

*2.* Isolate genomic *P. pacificus* DNA by growing 30 plates of the wild-type strain PS312 until nearly starved.

*3.* Wash worm plates with M9 buffer and pool in a 15ml falcon tube before cleaning by centrifugation at 800 x g and removal of excess M9. Repeat the washing with M9 a further three times to remove any remaining *E. coli* OP50.

*4.* Transfer worms to a 1.5ml microcentrifuge tube for DNA extraction (NEB Monarch Genomic DNA Purification Kit) following the manufactures protocol for animal tissue with minor modifications described below.

*5.* Add 250 µl of Tissue Lysis buffer with 15 µl proteinase K was to the worm pellet.

*6.* Incubate at 56°C for 4 h and agitate at 1400 rpm before centrifuging to pellet any remaining worm debris.

*7.* Remove supernatant and transfer to a clean 1.5 ml microcentrifuge tube.

*8.* Add 5 µl of RNase A to this lysate, vortex and incubated for 10 min at 56°C.

*9.* Bind and wash on a column as per the manufacturer’s instructions.

*10.* Elute DNA using TE buffer (Promega) as this leads to a higher transgenic efficiency in *P. pacificus* over other buffers.

*11.* Linearise the plasmid (pZH009) by digestion with restriction enzymes (we used NEB PstI-HF) for 1 h and digest the genomic DNA using the same restriction enzyme.

*12.* Clean digest using a MinElute PCR purification kit (Qiagen) as per the manufacturer’s guidelines but elute DNA in TE buffer.

*13.* Mix and dilute the linearised plasmid together with the genomic DNA with TE buffer to a final concentration of 10 ng/µl for linearised plasmid ( *Ppa-egl-20p* ::TurboRFP) and 60 ng/µl of genomic DNA.

*14.* Centrifuge the injection mix for 10 min at 4°C and 15000 x g and subsequently keep the mix on ice.

*15.* Microinject animals as described previously (Nakayama et al. 2020; Hiraga et al. 2021). We used a Zeiss Axiovert 200 together with Eppendorf microinjector.

*16.* Screen and isolate a strain carrying a complex array expressing desired marker ( *Ppa-egl-20p* ::TurboRFP).

*UV irradiation and screening*

Protocol used is based on previous methods for *C. elegans* with minor modifications (Mariol et al. 2013).

1. Pick 20 animals carrying the complex array to at least 10 separate culture plates.

2. Remove plate lids and irradiate using a UV Crosslinker (we use a CL-3000 from Analytik Jena) at a wavelength of 254 nm at 0.050 J / cm ^2^ .

3. After irradiation, place plates in 20°C incubator until progeny is old enough to screen for fluorescence (3-4 days).

4. Single out F1 fluorescent animals onto 100-250 individual culture plates.

5. Allow these F1 animals to propagate for a further 3-4 days until the progeny reaches an appropriate stage to screen the F2 for the presence of fluorescence.

6. Screen worms for successful integration events on a Zeiss Axio Zoom v.16 or equivalent microscope by observing an increase in number of fluorescent animals to ≥75% of the population.

7. From lines with ≥75 % fluorescence, pick 16 individual fluorescent worms from this population and after 3-4 days screen for plates with 100% fluorescence. This should be observed in roughly 33 % of these plates.

8. Select lines with 100% transmission and outcross with the original wild type strain to remove any additional mutations which may be generated by the UV irradiation.

9. After outcrossing, re-isolate animals with 100% transmission to confirm inheritance and integration of the transgene.

## Reagents

**Table:** 

**Strain**	**Genotype**	**Description / Availability**
PS312	Wildtype	Sommer lab
JWL19	*Ppa-dpy* ( *bnn13 [bnnEx5* [ *Ppa-egl-20p* ::TurboRFP]])	Uncloned *dpy* mutant generated in the strain carrying complex array *bnnEx5* [ *Ppa-egl-20p* ::TurboRFP] (JWL lab - this study)
JWL36	*bnnEx5* [ *Ppa-egl-20p* ::TurboRFP]	Strain carrying extrachromosomal array of *Ppa-egl-20p* ::TurboRFP (Gene ID PPA11519 and WBGene00101073) (JWL lab - this study)
JWL37	*bnnIs* 2[ *Ppa-egl-20p* ::TurboRFP]	Strain carrying integrated *Ppa-egl-20p* ::TurboRFP (Gene ID PPA11519 and WBGene00101073) (JWL lab - this study)
		
**Plasmids**	**Genotype**	**Description**
pZH009	*Ppa-egl-20p* ::TurboRFP:: *rpl-23* 3’UTR	2137 bp upstream region of *Ppa-egl-20* (Gene ID PPA11519 and WBGene00101073) predicted start site driving expression of TurboRFP codon optimised for *P. pacificus* and containing three *P. pacificus* introns from *Ppa-rps-1* in a pUC19 vector.
		
**Reagents**		
MiniPrep Kit	Qiagen (Cat#27104)	Plasmid purification.
MinElute PCR Purification Kit	Qiagen (Cat#28004)	Post restriction enzyme clean-up.
Monarch Genomic DNA Purification Kit	NEB (Cat#T3010)	*P. pacificus* genomic extraction.
PstI-HF	NEB (Cat#RS3140)	Restriction enzyme for plasmid and *P. pacificus* genomic extraction.
TE buffer	Promega (Cat#V6231)	Dilution of injection mix
